# One-year longitudinal association between changes in aortic regional morphology and muscle mass in cancer

**DOI:** 10.1038/s41598-025-06189-1

**Published:** 2025-07-01

**Authors:** Lu Gao, Wenjun Liu, Zixuan Meng, Jie Zheng, Lele Cheng, Like Ma, Yuan Gao, Tianyi Zhang, Yue Wu, Zhijie Jian

**Affiliations:** 1 Department of Radiology, The First Affiliated Hospital of Xi’an Jiaotong University, Xi’an, 710061 China; 2 Department of Cardiovascular Medicine, The First Affiliated Hospital of Xi’an Jiaotong University, Xi’an, 710061 China; 3 Biobank, The First Affiliated Hospital of Xi’an Jiaotong University, Xi’an, 710061 China

**Keywords:** Aortic morphology, Skeletal muscle, Physical activity, Computer tomography, Computed tomography, Nutrition

## Abstract

Cancer confers the risk for cardiovascular diseases. This longitudinal study aimed to explore the relationship between changes in aortic morphology and muscle mass in cancer. One hundred patients with cancer who underwent thoracoabdominal enhanced computed tomography (CT) at baseline and 1-year follow-up were retrospectively included. Aortic diameter and tortuosity were assessed using CT. Skeletal muscle mass was also evaluated. Pearson correlation analysis and multivariate-adjusted regression models were used to explore the relationship between changes in aortic morphology and muscle mass. Thirty-six patients were male. The average patient age was 57.3 ± 11.3 years. Breast cancer was the most common malignancy. A significant increase in aortic diameter and tortuosity and a decrease in muscle mass were observed. Aortic morphological changes were correlated with changes in muscle mass, diastolic pressure, and high-density lipoprotein cholesterol and C-reactive protein levels. In the fully adjusted multivariable regression analysis, a one-unit increase in muscle mass change was associated with a decrease of 0.503, 0.382, 0.241, 0.174, and 0.163 mm in the diameter of the L1-L5 aortic segments, respectively; a 0.020 decrease in aortic tortuosity; a 0.017 decrease in descending thoracic tortuosity; and a 0.016 decrease in abdominal aortic tortuosity. Increased muscle mass helps maintain aortic morphology in cancer, suggesting that muscle loss is a key factor in increased cardiovascular risk. Muscle might be an important therapeutic target to improve cancer patients’ prognosis.

**Supplementary Information:**

The online version contains supplementary material available at 10.1038/s41598-025-06189-1.

## Introduction

Cardiovascular disease (CVD) and cancer are the leading causes of death globally^1^. Improvements in technological advancements and cancer treatments have resulted in an overall decline in the cancer-related mortality rate, leading to more patients surviving longer and likely developing a secondary chronic disease. A study showed that 25% of patients with cancer had CVD, and the rate of first hospitalization for CVD in these patients was 30% higher than that in the general population^2^. Moreover, the CVD mortality risk is the highest within the first year after cancer diagnosis^3^. The increased CVD burden not only decreases the quality of life but also increases the cost of care in cancer survivorship^4^. Therefore, it is crucial to prevent CVD in patients with cancer.

Aortic morphological changes, such as tortuosity and dilation, are closely related to the occurrence and development of cardiovascular disease. These variations induce changes in haemodynamic processes, thereby increasing the burden on cardiovascular system and risk of cardiovascular disease. It is reported that the intricate geometrical features of aorta have prominent effects on haemodynamics^5^. What’s more, irregular contour and large degree of curvature of aorta might affect flow variables and wall shear stress, which is related to atheroma and aneurismal diseases^6^. Morphological changes in the aorta are also related to hypertension, aortic dissection and heart failure^3^. In addition, aortic dilation and tortuosity are closely associated with vascular remodeling, inflammation and oxidation that play a vital role in pathogenesis of cardiovascular disease^7^. Previous studies have indicated that arterial stiffness increases in patients with cancer^8^. Hence, accurate evaluations of aortic dilation and tortuosity are of great significance in predicting, preventing, and treating CVD in patients with cancer. Developments in imaging technology have facilitated comprehensive and systematic evaluation of aortic morphology. The overlap between cancer and CVD provides opportunities to assess both diseases concurrently through the use of computed tomography (CT), and CT is convenient in this context, with its 3D capabilities permitting reproducible and accurate measurements^9^.

Skeletal muscle is considered a biomechanical and endocrine organ, which plays important roles in health. Several studies revealed the prevalence of low muscle mass in patients with cancer^10–12^. What’s more, low muscle mass is related to cancer-related mortality^13^. Ko et al.^14^ showed that low muscle mass is an independent risk factor for coronary heart disease. Meanwhile, other research works suggested that lower skeletal muscle mass was related to metabolic syndrome, atherosclerotic CVD, heart failure, myocardial infarction, and atrial fibrillation^15,16^. Our previous study also found an association between skeletal muscle index and aortic diameter and tortuosity^17^. Hence, we hypothesized that aortic morphological changes were related to lower muscle mass in patients with cancer.

## Materials

This study was reviewed and approved by the ethical committee of the First Affiliated Hospital of Xi’ an Jiaotong University. All methods were performed in accordance with the relevant guidelines and regulations. Written informed consent was obtained from all participants.

### Subjects

The study included patients with cancer who underwent thoracoabdominal enhanced CT for disease evaluation between April 2022 and June 2024. The exclusion criteria were as follows: (1) patients with diabetes, hypertension, or kidney disease; (2) patients with dissection, aneurysms, vascular malformation, or any variations on CT; (3) patients whose CT images showed artifacts; (4) patients for whom the follow-up duration was less than 1 year; (5) patients for whom the life expectancy was less than two years; and (6) patients whose complete clinical information was not available for analysis.

### Characteristic collection

Information on the variables of interest such as age, sex, height, weight, systolic pressure, and diastolic pressure was collected. The primary tumor origin was also recorded. Peripheral blood was obtained with the patients in the supine position within 24 h of admission. Levels of triglycerides, low- and high-density lipoprotein cholesterol (LDL-C and HDL-C, respectively), total cholesterol, total protein, aspartate transaminase, alanine aminotransferase, alkaline phosphatase, urea, creatinine, hemoglobin, fasting glucose, and C-reactive protein (CRP) as well as red blood cell, white blood cell, and platelet counts were determined. Biochemical indicators were determined using standard approaches.

### CT image acquisition and evaluation of aortic diameter and tortuosity index

Thoracoabdominal enhanced CT data were collected at baseline and 1-year follow-up using two 256-slice CT scanners: Revolution CT (GE Healthcare, Milwaukee, WI) and Philips Brilliance iCT (Medical System, Best, The Netherlands). The parameters of CT scanning are presented in Supplementary Table 1. All patients accepted a standardized median cubital vein injection by power injector consisting of iomeprol with either 350 or 400 mg I/mL at a dose of 0.5 g I/kg and a rate of 2.7 mL/s follow by 30 mL saline chaser. CT was performed from lung apices to the iliac crest, without electrocardiography gating. All data were analyzed using a standard post-processing workstation (uInnovation-CT, R001, United Imaging Healthcare, Shanghai, China). The outer edge-to-outer edge method was used to measure the aortic diameter at five levels, as described previously^18^: ascending aorta (L1), located 1 cm distal to the aortic valve; descending aorta (L2), in line with L1; the level of the diaphragm (L3); 3 cm above the level of aortic bifurcation (L5); and midway between L3 and L5 (L4). Each segment of the aorta was defined by two points corresponding to the anatomical landmark. An automated centerline between the two points was generated. The tortuosity index of the aorta was calculated using the following formula: tortuosity index = centerline length/straight-line length. The aortic diameter and tortuosity were quantitatively analyzed at baseline and 1-year follow-up. Moreover, to account for the effect of diverse body shapes on data analysis, the diameter and tortuosity were standardized by body surface area (BSA). BSA was calculated using the formula^19^: BSA (m^2^) = 0.007184 × height (cm)^0.725^ × weight (kg)^0.425^.

### Evaluation of muscle mass using CT scans

The cross-sectional area of the mid-third lumbar (L3) vertebral muscle was considered as a proxy for muscle mass. 3D-Slicer software (Version 5.0.3, https://www.slicer.org/) was used to generate axial and sagittal CT images. Midline sagittal images were applied to determine the L3 level. Tissues with attenuation thresholds ranging from − 29 to 150 HU were automatically outlined on the corresponding axial image. The paraspinal, psoas, and abdominal wall muscles were manually delineated. After that, muscle mass can be automatically obtained. Muscle mass was quantitatively analyzed at baseline and 1-year follow-up. Finally, muscle mass was standardized by BSA.

The intraobserver and interobserver reliability were evaluated for aortic diameter, tortuosity index and muscle mass. Two experienced radiologists (L.G. and Z.J.J., both with 6 years of experience in cardiovascular imaging) perfomed the measurements independently and the time interval of the two repeated measurements for assessing the intraobserver reliability was 3 months. Intraclass correlation coefficients (ICC) and Bland-Altman plots were used to evaluate the intraobserver and interobserver reliability.

### Statistical analysis

Data analysis was conducted using SPSS version 23.0 software (IBM Corp, Armonk, NY, USA) and MedCalc (MedCalc 13.0, Mariakerke, Belgium). ICC and Bland-Altman plots were used for consistency. Results are presented as mean ± standard deviation or number (percentage), as appropriate. Differences between baseline and 1-year follow-up were assessed using paired t-tests or Wilcoxon signed rank test according to whether the data showed normal distribution. Pearson correlation analysis was used to analyze the relationship between the changes in morphological features and those in the potential influencing factors. Multivariate linear regression analysis was performed to assess if the change in muscle mass was associated with changes in the morphological characteristics of the aorta. Age, sex, and body mass index (BMI) were adjusted for model 1, and age, sex, BMI, changes in HDL-C level, diastolic pressure, and CRP were adjusted for model 2. Linear regression coefficient and the 95% confidence interval (CI) have been reported. *P* < 0.05 was considered statistically significant.

## Results

**Fig. 1:**
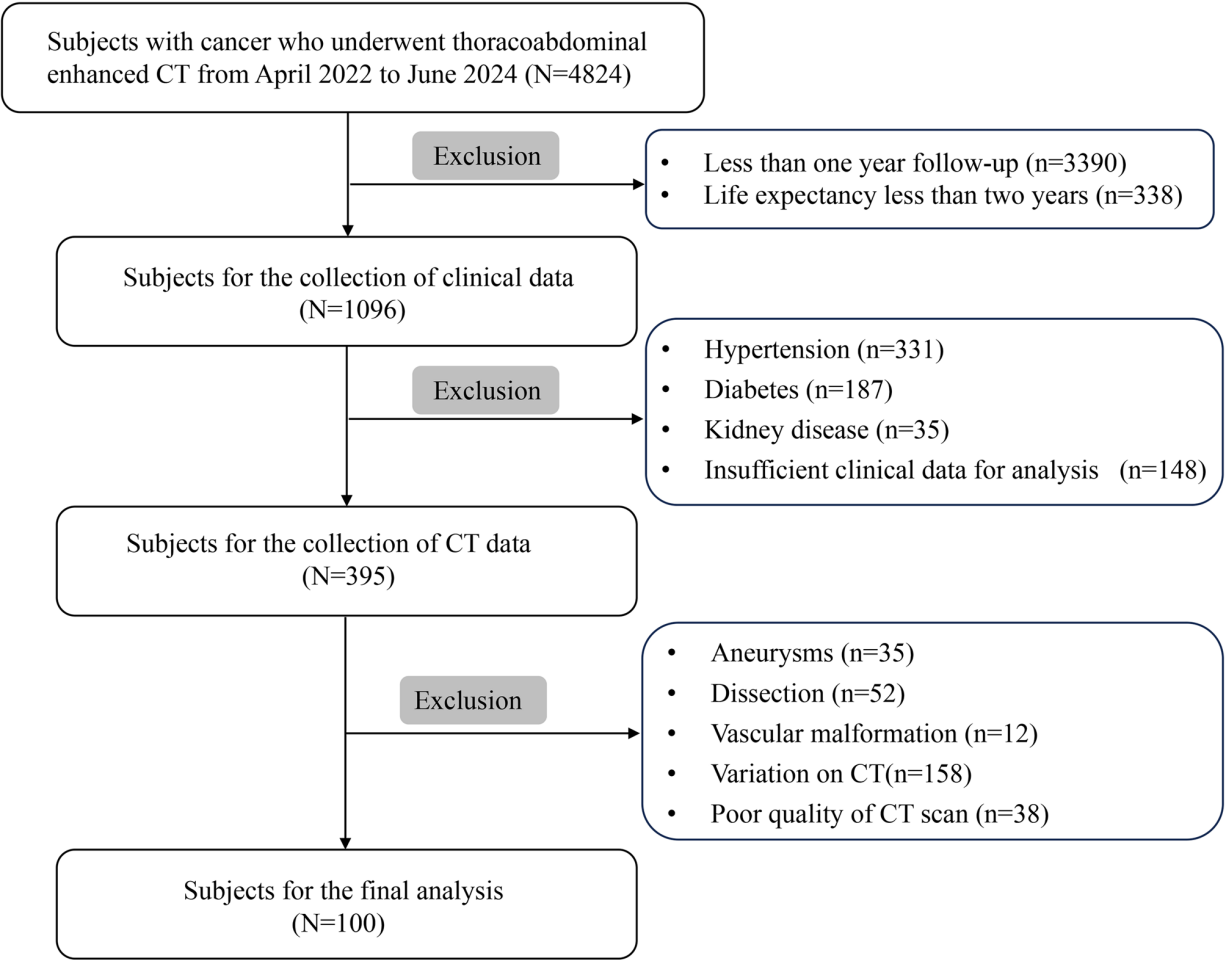
Flowchart of the screening process. *CT* computed tomography.

The study flowchart is shown in Fig. 1. Overall, 100 patients (64 women and 36 men) were enrolled in this study; the average patient age was 57.3 ± 11.3 years. All patients had malignant tumors. Most tumors were those of the breast (36%) and lungs (32%). Additionally, a considerable number of tumors were urinary carcinomas/carcinomas of the reproductive system (22%). The detailed baseline demographic information and clinical characteristics of the patients are presented in Table 1.

**Table 1:** Baseline characteristics of the participants.

Age, year	57.3 ± 11.3
Male, %	36 (36)
BMI, kg/m^2^	23.8 ± 3.2
Smoking, %	31 (31)
Systolic pressure, mmHg	119.0 ± 14.1
Diastolic pressure, mmHg	78.2 ± 8.8
Triglycerides, mg/dL	1.6 ± 0.9
HDL-C, mmol/L	1.2 ± 0.3
LDL-C, mmol/L	2.8 ± 0.8
Total cholesterol, mmol/L	4.6 ± 0.9
Total protein, g/L	71.7 ± 5.4
Aspartate transaminase, U/L	23.4 ± 10.5
Alanine aminotransferase, U/L	20.2 ± 13.4
Alkaline phosphatase, U/L	114.8 ± 137.9
Urea, mmol/L	4.9 ± 1.3
Creatinine, µmol/L	57.7 ± 14.3
Hemoglobin, g/L	129.4 ± 19.0
Fasting glucose, mmol/L	5.2 ± 1.1
CRP, mg/L	22.4 ± 16.0
Red blood cell count	4.3 ± 0.6
White blood cell count	6.4 ± 2.6
Platelet count	261.2 ± 91.6
Primary tumor site, %	
Lung	32 (32)
Digestive system	6 (6)
Urinary/reproductive system	22 (22)
Breast	36 (36)
Other	4 (4)
Cancer status, %	
Localised	64 (64)
Locally advanced/advanced	36 (36)
Metastatic/recurrent	0 (0)
Treatment, %	
Single modality	
Surgery only	49 (49)
Chemotherapy	18 (18)
Radiation	12 (12)
Multiple modalities	
Surgery ± radiation and/ or chemotherapy	18 (18)
Radiation and chemotherapy	3 (3)

All results were reported as mean ± standard deviations or number (percentage).

*BMI* Body mass index, *HDL-C* high-density lipoprotein cholesterol, *LDL-C* low-density lipoprotein cholesterol, *CRP* C-reactive protein.

The intraobserver reliability for measuring aortic diameters, tortuosity index and muscle mass was excellent, with values ranging from 0.880 to 0.989. In terms of interobserver reliability, the measurement of aortic diameters, tortuosity index and muscle mass also exhibited excellent values ranging from 0.868 to 0.984 (Supplementary Table 2).

The Bland-Altman plots indicated that the differences between the two repeated measurements fell within the limits of agreement (Supplementary Fig. 1A and 1B).

**Fig. 2:**
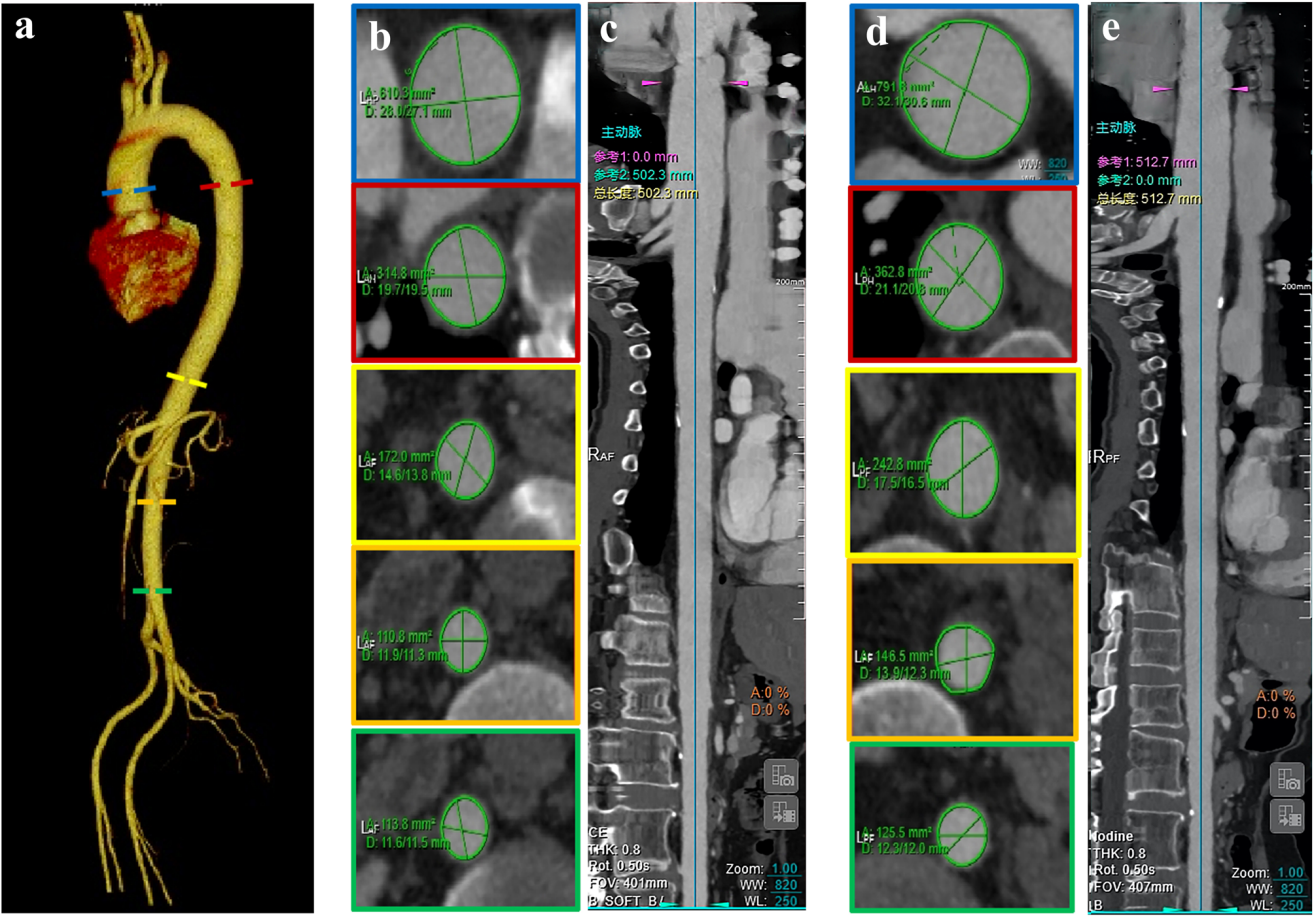
Morphological changes in the aorta of (**a**) 35-year-old female with lung cancer at baseline (**b,c**) and the 1-year follow-up (**d,e**).

Quantitative changes in the regional morphology of the aorta are shown in Table 2. The aortic diameter and tortuosity were increased (L1: from 18.671 mm to 20.123 mm, percent change: 7.269%, *p* = 0.001; L2: from 12.791 mm to 14.101 mm, percent change: 10.371%, *p <* 0.001; L3: from 11.822 mm to 13.001 mm, percent change: 10.149%, *p <* 0.001; L4: from 9.015 mm to 10.074 mm, percent change: 12.250%; and L5: from 8.955 mm to 9.816 mm, percent change: 9.974%, *p <* 0.001; aortic tortuosity: from 1.152 to 1.195, percent change: 5.775%, *p* = 0.002; tortuosity of the descending thoracic aorta (DTA): from 1.135 to 1.183, percent change: 6.889%, *p <* 0.001; and tortuosity of the ascending aorta: from 0.663 to 0.696, percent change: 5.082%, *p* = 0.014) (Fig. 2).

**Table 2:** Longitudinal changes in aortic regional morphology.

	Baseline	Follow-up	Change (%)	*p* value
L1 (mm)	18.671 ± 2.891	20.123 ± 3.073	7.269 ± 9.098	0.001
L2 (mm)	12.791 ± 1.601	14.101 ± 1.930	10.371 ± 8.969	< 0.001
L3 (mm)	11.822 ± 1.659	13.001 ± 1.839	10.149 ± 6.400	< 0.001
L4 (mm)	9.015 ± 1.513	10.074 ± 1.599	12.250 ± 9.716	< 0.001
L5 (mm)	8.955 ± 1.383	9.816 ± 1.543	9.974 ± 10.000	< 0.001
Aorta tortuosity	1.152 ± 0.140	1.195 ± 0.200	5.775 ± 3.071	0.002
DTA tortuosity	1.135 ± 0.195	1.183 ± 0.266	6.889 ± 3.806	< 0.001
AA tortuosity	0.663 ± 0.138	0.696 ± 0.137	5.082 ± 4.519	0.014

All results were reported as mean ± standard deviations.

*DTA* descending thoracic aorta, *AA* abdominal aorta.

**Fig. 3:**
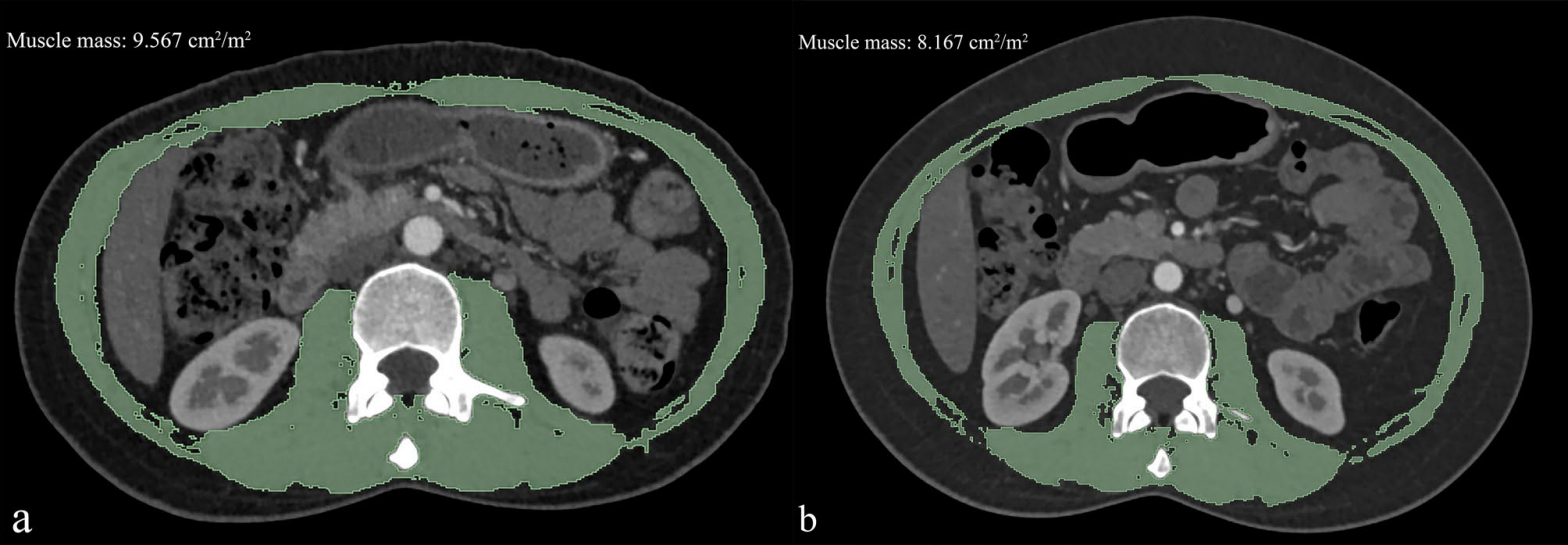
Muscle mass assessment of a 35-year-old female with lung cancer at baseline (**a**) and the 1-year follow-up (**b**).

Factors influencing aortic morphology at baseline and follow-up are shown in Table 3. Significant changes were found in HDL-C, LDL-C, systolic pressure, diastolic pressure, hemoglobin, and CRP. Moreover, muscle mass significantly decreased (Fig. 3). However, BMI and the levels of total cholesterol, triglycerides, and fasting glucose did not change.

**Table 3:** Longitudinal changes in muscle mass and risk factors.

Variables	Baseline	Follow-up	*p* value
Total cholesterol, mmol/L	4.649 ± 0.897	4.573 ± 1.174	0.430
HDL-C, mmol/L	1.239 ± 0.311	1.256 ± 0.301	0.032
LDL-C, mmol/L	2.829 ± 0.790	2.935 ± 0.752	< 0.001
Systolic pressure, mmHg	118.961 ± 14.100	114.070 ± 12.876	< 0.001
Diastolic pressure, mmHg	78.177 ± 8.760	74.220 ± 7.956	0.003
Triglycerides, mmol/L	1.640 ± 0.863	1.632 ± 0.802	0.867
Fasting glucose, mmol/L	5.246 ± 1.066	5.206 ± 1.200	0.554
Hemoglobin, mmol/L	129.356 ± 19.000	120.720 ± 17.368	0.029
CRP, mg/L	16.444 ± 14.109	22.474 ± 15.176	< 0.001
BMI, kg/m^2^	23.799 ± 3.203	23.633 ± 3.528	0.353
Muscle mass, cm^2^/m^2^	6.899 ± 1.341	5.877 ± 1.195	< 0.001

All results were reported as mean ± standard deviations.

*BMI* Body mass index, *HDL-C* high-density lipoprotein cholesterol, *LDL-C* low-density lipoprotein cholesterol, *CRP* C-reactive protein.

**Fig. 4:**
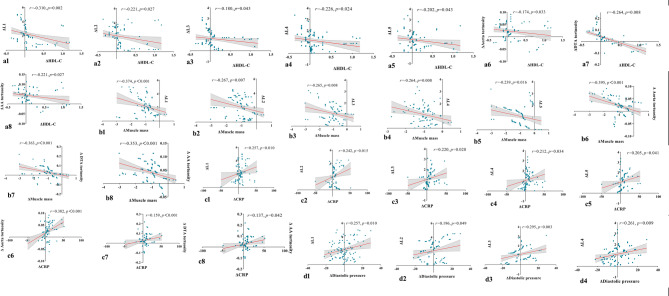
Analysis of correlations between aortic morphological changes and variations in the potential influencing factors (**a1–d4**).

The correlations between aortic morphology and influencing factors are shown in Fig. 4. The changes in L1-L5 and tortuosity of the aorta, DTA, and AA were significantly correlated with the change in HDL-C (*r* = − 0.310, *p* = 0.002; *r* = − 0.221, *p* = 0.027; *r* = − 0.180, *p* = 0.043; *r* = − 0.226, *p* = 0.024; *r* = − 0.202, *p* = 0.043; *r* = − 0.174, *p* = 0.033; *r* = − 0.264, *p* = 0.008; *r* = − 0.221, *p* = 0.027, respectively) and muscle mass (*r*= − 0.347, *p <* 0.001; *r* = − 0.267, *p* = 0.007; *r* = − 0.265, *p* = 0.008; *r* = − 0.264, *p* = 0.008; *r* = − 0.239, *p* = 0.016; *r* = − 0.395, *p <* 0.001; *r* = − 0.363, *p <* 0.001; *r* = − 0.353, *p <* 0.001, respectively). We also found positive correlations between the change in CRP levels and those in aortic morphology (*r* = 0.257, *p* = 0.010; *r* = 0.242, *p* = 0.015; *r* = 0.220, *p* = 0.028; *r* = 0.212, *p* = 0.034; *r* = 0.205, *p* = 0.041; *r* = 0.382, *p <* 0.001; *r* = 0.195, *p <* 0.001; *r* = 0.173, *p* = 0.042). The change in diastolic pressure (L1-L4: *r* = 0.257, *p* = 0.010; *r* = 0.196, *p* = 0.049; *r* = 0.295, *p* = 0.003; *r* = 0.261, *p* = 0.009) only correlated with the change in the diameters of the proximal aortic segment. However, there was no remarkable relationship between the change in aortic morphology and that in systolic pressure (*r* = 0.128, *p* = 0.205; *r* = 0.067, *p* = 0.510; *r* = 0.164, *p* = 0.104; *r* = 0.014, *p* = 0.894; *r* = 0.138, *p* = 0.172; *r* = 0.139, *p* = 0.169; *r* = 0.137, *p* = 0.174; *r* = 0.192, *p* = 0.056), hemoglobin (*r* = 0.103, *p* = 0.307; *r* = 0.101, *p* = 0.320; *r* = 0.119, *p* = 0.237; *r* = 0.164, *p* = 0.102; *r* = 0.073, *p* = 0.473; *r* = 0.163, *p* = 0.106; *r* = 0.016, *p* = 0.877; *r* = 0.095, *p* = 0.349) and LDL-C (*r* = 0.232, *p* = 0.406; *r* = 0.247, *p* = 0.277; *r* = 0.257, *p* = 0.430; *r* = 0.091, *p* = 0.366; *r* = 0.076, *p* = 0.451; *r* = 0.133, *p* = 0.188; *r* = 0.243, *p* = 0.108; *r* = 0.160, *p* = 0.077).

In the fully adjusted multivariable regression analysis, a one-unit decrease in muscle mass change was associated with an increase of 0.503, 0.382, 0.241, 0.174, and 0.163 mm in the L1-L5 diameter respectively, a increase of 0.020 in aortic tortuosity, a increase of 0.017 in DTA tortuosity, and a increase of 0.016 in AA tortuosity. Detailed results of the regression analysis are shown in Table 4.

**Table 4:** Multiple linear regression analysis between the changes of muscle mass and aortic morphology.

	Model	β (95% CI)	*p* Value
ΔL1	1	− 0.670 (− 1.209, − 0.131)	0.015
	2	− 0.503 (− 1.046, 0.040)	0.039
ΔL2	1	− 0.500 (− 0.896, − 0.103)	0.014
	2	− 0.382 (− 0.739, − 0.025)	0.036
ΔL3	1	− 0.379 (− 0.633, − 0.124)	0.004
	2	− 0.241 (− 0.439, − 0.043)	0.018
ΔL4	1	− 0.360 (− 0.558, − 0.163)	< 0.001
	2	− 0.174 (− 0.363, 0.015)	0.041
ΔL5	1	− 0.349 (− 0.588, − 0.111)	0.005
	2	− 0.163 (− 0.464, − 0.102)	0.003
ΔTortuosity _aorta_	1	− 0.032 (− 0.039, − 0.024)	< 0.001
	2	− 0.020 (− 0.030, − 0.014)	< 0.001
ΔTortuosity _DTA_	1	− 0.024 (− 0.032, − 0.017)	< 0.001
	2	− 0.017 (− 0.023, − 0.012)	< 0.001
ΔTortuosity _AA_	1	− 0.022 (− 0.038, − 0.006)	0.006
	2	− 0.016 (− 0.027, − 0.006)	0.002

Model 1 was adjusted for age, gender and BMI; model 2 was adjusted for age, sex, BMI, ΔHDL-C, Δdiastolic pressure and ΔCRP.

*Δ* change, *DTA* descending thoracic aorta, *AA* abdominal aorta; *BMI* Body mass index, *HDL-C* high-density lipoprotein cholesterol, *CRP* C-reactive protein.

## Discussion

Cancer survivors are at increased risk of CVD as well as death from CVD^20^. Aortic morphology has been found to be the predictor of CVD events^21^. Additionally, previous research has shown that low muscle mass is associated with an increased risk of cardiovascular related mortality^22^. However, the data available were limited, mainly pertained to aortic diameter, and suggested an association of aortic diameter with influencing factors^23^. The evidence on tortuosity in cancer remains limited. The relationship between long-term changes in aortic morphology and those in muscle mass in cancer remains unknown. For the first time, we conducted a 1-year longitudinal study on cancer patients, which revealed a negative correlation between changes in muscle mass and morphological remodeling of the aorta, including dilation and tortuosity. We conducted a comprehensive analysis of the morphological features of various segments of the aorta and muscle mass, thereby providing novel imaging biomarkers for assessing cardiovascular risk in cancer patients.

Through longitudinal study, we found that the diameter and tortuosity of the aorta increased over one year in cancer patients. These morphological changes are associated with the aging process and progressive arterial stiffness. Imaging confirmed that the aorta in cancer patients underwent significant adverse remodeling within a year following diagnosis and treatment. Importantly, this phenomenon may be associated with the increased incidence of CVD and poor cardiovascular prognosis in cancer patients^24^. Concurrently, we observed a significant decrease in muscle mass among cancer patients over one year, indicating that these patients are at a higher nutritional risk and more prone to muscle loss^25^. Cancer patients have a significantly increased risk of cardiovascular complications, which has an important impact on the overall prognosis of patients. In the context of cardiovascular risk assessment, recent studies have highlighted two key dimensions. First, traditional risk factors for cardiovascular disease, such as hypertension, diabetes and obesity, are also applicable in cancer patients, and these factors are closely related to cardiovascular complications related to cancer treatment^26^. Second, emerging evidence suggests that cancer treatments themselves may induce cardiovascular toxicity. For instance, the CARDIOTOX registry study demonstrated that pre-treatment cardiovascular risk evaluation using the SCORE system effectively predicts severe cardiotoxicity and all-cause mortality during cancer treatment, primarily through traditional risk factor profiling^27^. In our study, we identified a negative correlation between changes in muscle mass and alterations in aortic morphology. Even after controlling for confounding variables, higher muscle mass was significantly associated with reduced aortic diameter and tortuosity. These findings indicate that muscle loss is associated with inadequate morphological remodeling of the aorta. Furthermore, a randomized controlled trial demonstrated that long-term physical activity can reduce CVD risk in cancer survivors^28^. This suggested that muscle mass may serve as a potential cardiovascular risk factor for future interventions in cancer patients. Such implications may extend across age groups. While comprehensive geriatric assessments (incorporating frailty, nutrition, and cognitive evaluations) have proven effective for cardiovascular risk identification in elderly cancer patients (> 70 years)^29^. Our study focused on middle-aged to older adults (40–70 years), and this demonstrated that muscle mass preservation remains a critical concern independent of chronological age.

Contrast-enhanced CT is the primary modality utilized for evaluating tumor grade and patient prognoses, particularly in distinguishing changes following cancer treatment. In this study, we utilized contrast-enhanced CT data from the chest and abdomen to comprehensively assess the diameter and tortuosity of various segments of the aorta, evaluate skeletal muscle mass, and investigate the relationship between muscle mass and aortic morphological remodeling. This approach offers a novel method for assessing cardiovascular risk without necessitating additional procedures for patients. Furthermore, our segmental analysis revealed a stronger association between the changes in the diameter of proximal aortic segments and muscle mass. Additionally, compared with the changes in thoracic aorta tortuosity, the changes in abdominal aorta tortuosity showed a weaker association with muscle mass changes. The preferential influence of muscle mass on the proximal segment of the aorta may be related to the structure of the aorta. Segmental changes exist in the structure, composition, and hemo- dynamics of the aorta^30,31^. Significant differences were found in morphometry and composition among different segments of the aorta, with the collagen and elastin contents, respectively, being higher distally and proximally^32^. Patients with cancer are markedly insulin resistant^33^. Insulin resistance would result in a decrease in elastin fibers, reducing the stretch and flexibility of the aorta and leading to changes in aortic tortuosity and dilatation^34^.

A previous study showed that HDL-C, diastolic pressure, and systolic pressure were associated with aortic diameter^23^. However, our current study indicated a link between aortic diameter and tortuosity and HDL-C, diastolic pressure, and CRP in some aortic segments. High HDL-C levels are positively correlated with a decreased cancer risk^35^. It is also generally accepted that HDL-C has many protective effects on the cardiovascular system. Hence, the improvement in HDL-C levels is good for cardiovascular health in cancer patients. A large prospective cohort study showed that hypertension was related to increased risks of cancer and mortality^36^. Our data indicated a positive correlation of diastolic pressure with the diameters of the aortic segments L1-L4. Surprisingly, systolic pressure was not related to morphological features. Excessive secretion of renin, angiotensin, and angiotensin I by carcinoma cells may lead to paraneoplastic hypertension^21^. Hypertension might result in the dysfunction of endothelial cells and vascular smooth muscles, further inducing morphological and physiological changes in the arterial wall^37^. We also found CRP was positively associated with aortic morphology. Previous reports have shown that inflammation plays a crucial role in tumorigenesis and vascular structure^38^. The soft inner elastic membrane degrades, and the tunica intima thickens owing to increased protein deposition in the basement membrane^39^. Additionally, increased collagen fiber deposition decreases the flexibility of the aorta, which ultimately results in aortic morphology changes^39^. Although we did not explore the relationship between aortic morphology and categorical variables, such as sex, these variables did not change during the follow-up period. However, sex differences in aortic morphology exist^23^. To eliminate the effect of sex on the relationship between aortic morphology and muscle mass, we adjusted for these factors as confounding factors. To explore the effect of categorical variables on aortic morphology, subgroup analysis is required.

After a 1-year longitudinal follow-up, we observed decreased muscle mass and increased aortic diameter and tortuosity in patients with cancer. The underlying mechanisms of this phenomenon may involve inflammation, endocrine and other factors. Previous research has indicated that cancer survivors may face a higher burden of low muscle mass^40^. Skeletal muscle is the main organ responsible for glycogen storage and glucose uptake, which play central roles in metabolic homeostasis^41^. Low muscle mass is related to an increased risk for the development of type 2 diabetes and insulin resistance^42^. However, improved skeletal muscle strength and physical activity are cornerstones in the prevention, treatment, and management of metabolic syndrome, type 2 diabetes mellitus, and hepatic steatosis^43^. Furthermore, proinflammatory cytokines, such as tumor necrosis factor-α, interleukin-1, and interleukin-6, secreted from tumors may cause appetite loss and contribute to the degradation of myofibrillar proteins and reduced protein synthesis, eventually resulting in muscle wasting^44,45^. Inflammatory states in cancer might prevent iron absorption by the gut and promote iron retention by macophages^46^. The resulting iron deficiency triggers anemia, which might affect skeletal muscle oxygenation and cardiac function^47^. Inflammation may lead to endothelial cell dysfunction and thus cause abnormal vasodilation and vasocontriction^39^. Our study also indicated that CRP was positively associated with aortic morphological characteristics, which suggests that inflammation might play an important role in aortic morphology. Moreover, recent studies suggested that the skeletal muscle is an endocrine organ and secretes a vast majority of myokines^48^. Some myokines such as fibroblast growth factor 21, musclin, and apelin have a salutary effect on the cardiovascular system, which is related to protection against atherosclerosis and blood pressure control. Hence, skeletal muscle atrophy may lead to impaired cardiovascular protection function^48^. Further, the “muscle pump” effect is crucial in regulating blood flow during exercise^49^. During muscle contraction, peripheral venous blood is expelled, thus promoting venous return and increasing cardiac output and stroke volume^49^. Low muscle mass will change the “muscle pump” effect, affect returned blood volume and aortic afterload, alter hemodynamics, and finally change the aortic morphology. Overall, we hypothesize that the specific characteristics of cancer, in conjunction with muscle loss, may adversely affect vascular morphology, thereby creating a vicious cycle involving cancer, muscle, and the aorta.

This study has several limitations. First, this was a single-center study. Multicenter studies with larger sample sizes were needed to verify our findings. Second, we did not evaluate skeletal muscle function, which might have potentially affected the relationships described herein. In addition, information regarding medication use and smoking habits is worth considering; however, unfortunately, we could not obtain these data. This limitation might have limited us from fully assessing the confounding factors in the correlation between muscle mass and the geometry of the aorta. Finally, different tumor types were included in this study, which may have affected the results to some extent. In the future, we would include patients with a specific tumor type to validate our results.

## Conclusions

Our study revealed the relationship between muscle loss and aortic regional morphological changes in cancer, highlighting that muscle loss is a key factor in increased cardiovascular risk. This study advanced the understanding of the mechanisms of cancer-cardiovascular comorbidity and promoted the development of comprehensive interventions for cancer patients. Muscle might be an important therapeutic target, with strategies such as nutritional support and physical therapy offering potential to improve cancer patients’ prognosis.

## Electronic supplementary material

Below is the link to the electronic supplementary material.

Supplementary Material 1

Supplementary Material 2

## Data Availability

The datasets used and/or analyzed during the present study are available from the corresponding author on reasonable request.
