# Supplementary material for: One-year longitudinal association between changes in aortic regional morphology and muscle mass in cancer
Source: Sci Rep. 2025 Jul 1;15:22130. doi: 10.1038/s41598-025-06189-1 (PMC12215070; doi:10.1038/s41598-025-06189-1)
Supplement: Supplementary file 1 — Supplementary Material 1 [file 41598_2025_6189_MOESM1_ESM.docx]

**Supplementary Table 1**. Parameters of thoracoabdominal enhanced CT scanning.

|  | Philips Brilliance iCT | GE Revolution CT |
| --- | --- | --- |
| Matrix size | 512×512 | 512×512 |
| Slice thickness, mm | 0.625 | 0.625 |
| Pitch, mm | 0.900 | 0.992 |
| Tube voltage, kV（depended on the patient’s weight） |  |  |
| <70 kg | 100 | 100 |
| ≥70kg | 120 | 120 |
| Tube current, mA（depended on the patient’s weight） |  |  |
| <70 kg | 300 | 300 |
| ≥70kg | 250 | 250 |

**Supplementary Table 2.** The Intraclass Correlation of Intra‐and interobserver reproducibility for the assessment of aortic diameters , tortuosity index and muscle mass.

| Variables | Intraobserver | | Interobserver | |
| --- | --- | --- | --- | --- |
|  | ICC | 95% CI | ICC | 95% CI |
| L1, mm | 0.989 | 0.984 to 0.993 | 0.984 | 0.977 to 0.989 |
| L2, mm | 0.941 | 0.914 to 0.960 | 0.923 | 0.887 to 0.947 |
| L3, mm | 0.946 | 0.921 to 0.963 | 0.945 | 0.919 to 0.963 |
| L4, mm | 0.954 | 0.932 to 0.969 | 0.942 | 0.915 to 0.961 |
| L5, mm | 0.947 | 0.922 to 0.964 | 0.945 | 0.919 to 0.963 |
| Aorta tortuosity | 0.954 | 0.932 to 0.969 | 0.948 | 0.924 to 0.965 |
| DTA tortuosity | 0.880 | 0.827 to 0.918 | 0.873 | 0.817 to 0.913 |
| AA tortuosity | 0.934 | 0.904 to 0.955 | 0.930 | 0.898 to 0.952 |
| Muscle mass | 0.916 | 0.877 to 0.942 | 0.868 | 0.811 to 0.910 |

Note: ICC, Intraclass correlation coefficient; CI: Confidence Interval; DTA, Descending thoracic aorta; AA, abdominal aorta
