# Supplementary material for: One-year longitudinal association between changes in aortic regional morphology and muscle mass in cancer
Source: Sci Rep. 2025 Jul 1;15:22130. doi: 10.1038/s41598-025-06189-1 (PMC12215070; doi:10.1038/s41598-025-06189-1)
Supplement: Supplementary file 2 — Supplementary Material 2 [file 41598_2025_6189_MOESM2_ESM.pptx]

## Slide 1
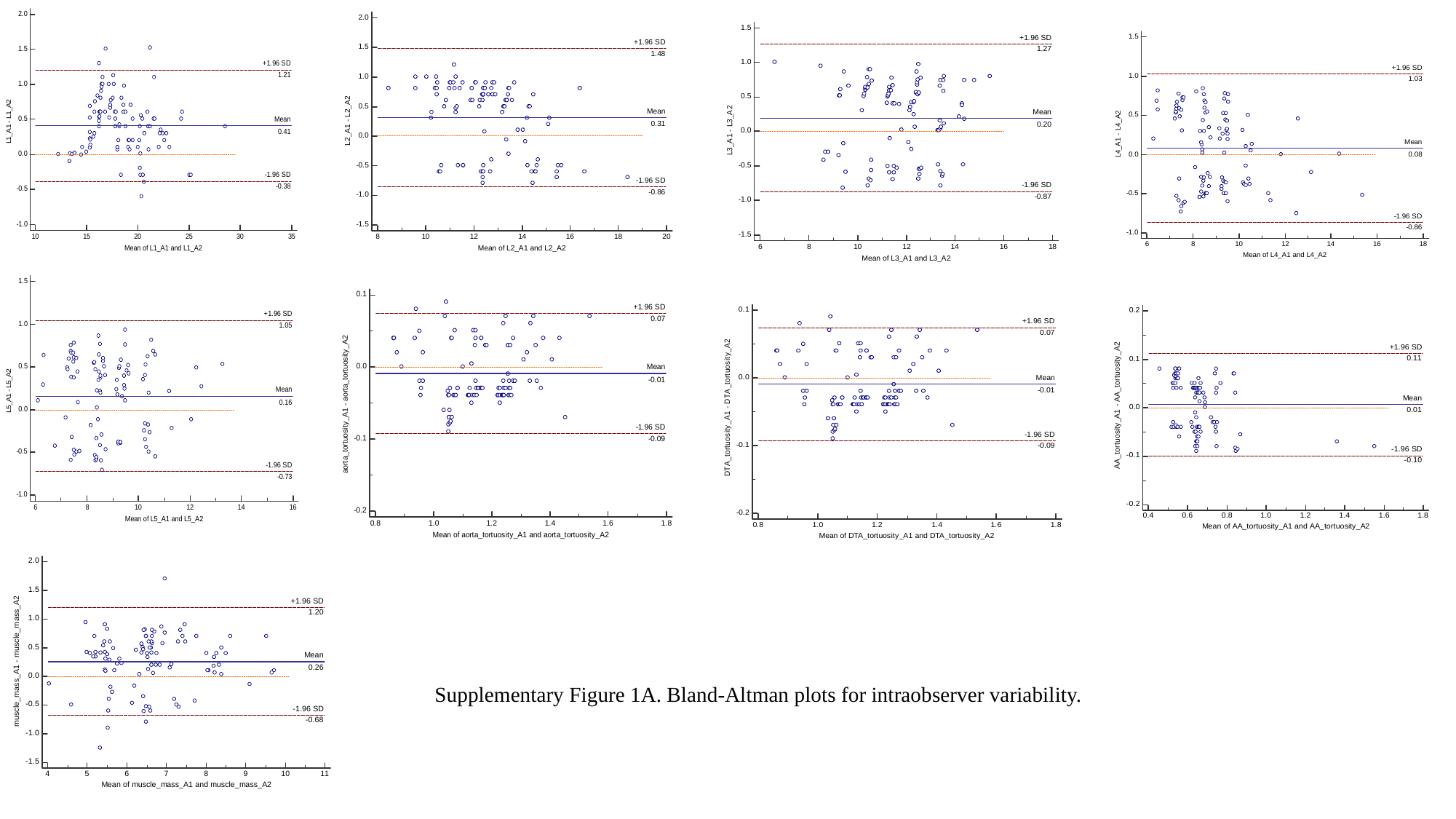

Supplementary Figure 1A. Bland-Altman plots for intraobserver variability.

## Slide 2
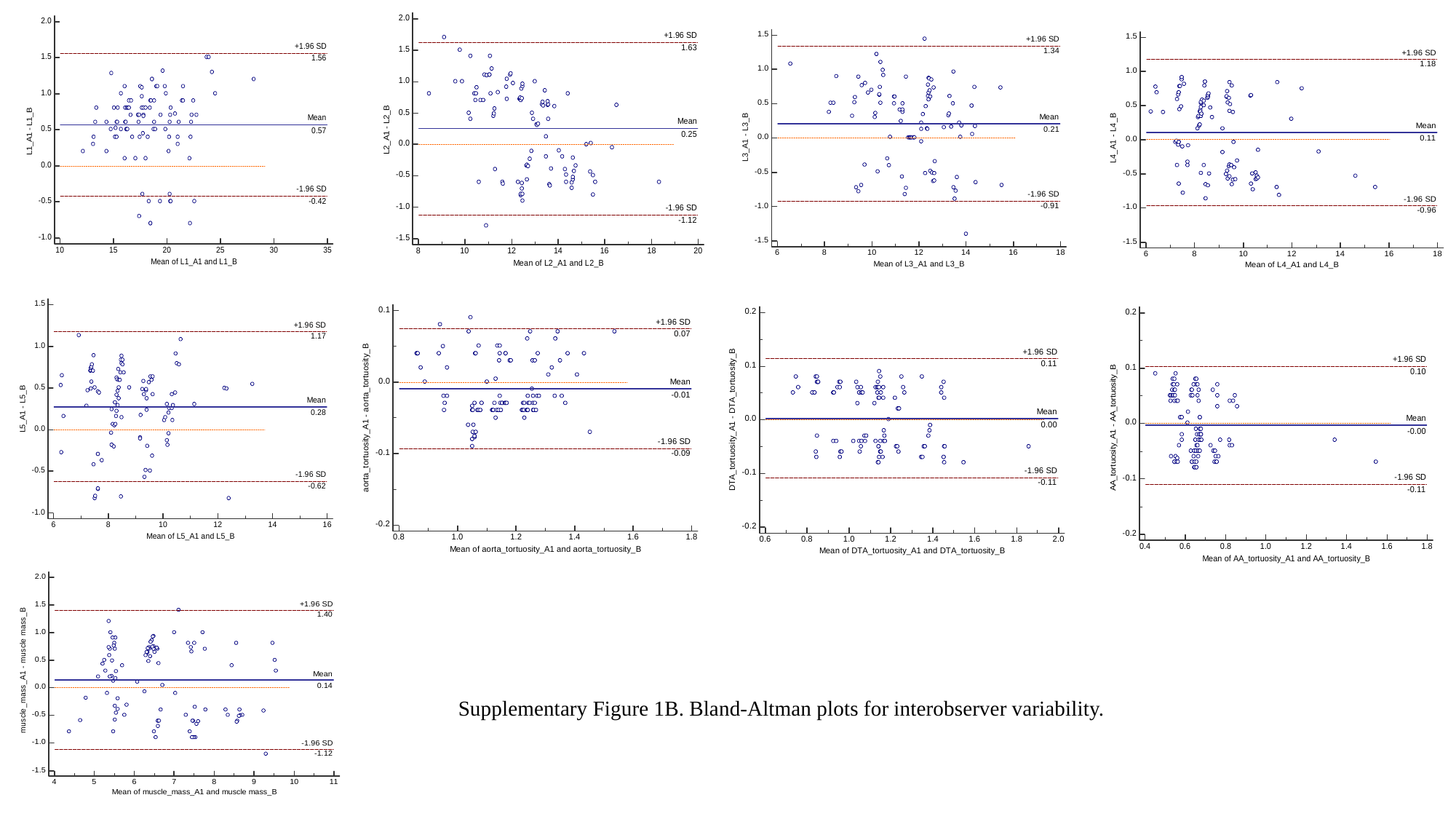

Supplementary Figure 1B. Bland-Altman plots for interobserver variability.
